# On‐Chip Optical Detection of Viruses: A Review

**DOI:** 10.1002/adpr.202000150

**Published:** 2021-02-25

**Authors:** Yuzhi Shi, Zhenyu Li, Patricia Yang Liu, Binh Thi Thanh Nguyen, Wenshuai Wu, Qianbin Zhao, Lip Ket Chin, Minggui Wei, Peng Huat Yap, Xiaohong Zhou, Hongwei Zhao, Dan Yu, Din Ping Tsai, Ai Qun Liu

**Affiliations:** ^1^ School of Electrical and Electronic Engineering Nanyang Technological University Singapore 639798 Singapore; ^2^ National Key Laboratory of Science and Technology on Micro/Nano Fabrication Institute of Microelectronics Peking University Beijing 100871 China; ^3^ Center for Systems Biology Massachusetts General Hospital Boston MA 02141 USA; ^4^ Lee Kong Chian School of Medicine Nanyang Technological University Singapore 308232 Singapore; ^5^ State Key Joint Laboratory of ESPC School of Environment Tsinghua University Beijing 100084 China; ^6^ State Key Laboratory of Marine Resource Utilization of South China Sea Hainan University Haikou 570228 China; ^7^ Beijing Pediatric Research Institute Beijing Children's Hospital Capital Medical University National Center for Children's Health Beijing 100045 China; ^8^ Department of Electronic and Information Engineering The Hong Kong Polytechnic University Hung Hom Kowloon Hong Kong China

**Keywords:** COVID‐19, optical detection, optofluidics, surface modifications, viruses

## Abstract

The current outbreak of the coronavirus disease‐19 (COVID‐19) pandemic worldwide has caused millions of fatalities and imposed a severe impact on our daily lives. Thus, the global healthcare system urgently calls for rapid, affordable, and reliable detection toolkits. Although the gold‐standard nucleic acid amplification tests have been widely accepted and utilized, they are time‐consuming and labor‐intensive, which exceedingly hinder the mass detection in low‐income populations, especially in developing countries. Recently, due to the blooming development of photonics, various optical chips have been developed to detect single viruses with the advantages of fast, label‐free, affordable, and point of care deployment. Herein, optical approaches especially in three perspectives, e.g., flow‐free optical methods, optofluidics, and surface‐modification‐assisted approaches, are summarized. The future development of on‐chip optical‐detection methods in the wave of emerging new ideas in nanophotonics is also briefly discussed.

## Graphical abstract

Optical methods for virus detection have the advantages of fast, label‐free, affordable, and point of care deployment. Herein, optical approaches in three perspectives, e.g., flow‐free optical methods, optofluidics, and surface‐modification‐assisted approaches, are summarized. The future development of on‐chip optical‐detection methods in the wave of emerging new ideas in nanophotonics is also briefly discussed.

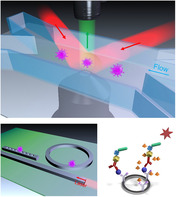

## 1 Introduction

Outbreaks of emerging infections occur more frequently and have more severe global impact in recent decades due to continuing population growth and urbanization.^[^ ^1^, ^2^ ^]^ Such viral outbreaks include Severe Acute Respiratory Syndrome (SARS) in 2003, H1N1 (swine flu) influenza in 2009, Middle East Respiratory Syndrome (MERS) in 2012, Zika in 2016, and the current coronavirus disease‐19 (COVID‐19) pandemic.^[^ ^3^, ^4^, ^5^, ^6^, ^7^ ^]^ In tropical countries such as Singapore, the mosquito breeding also leads to the spread of dengue disease, leading to 4.2 million cases worldwide in 2019.^[^ ^8^ ^]^

Up to the early October 2020, over 50 million people have been infected by SARS‐CoV‐2, and the mortality rate is 2.48%.^[^ ^9^ ^]^ The public health measures in global travel restrictions, social and economic activities limitations affect more than half of world populations.^[^ ^10^, ^11^ ^]^ Such measures had a huge global impact and caused severe economy recession. For instance, USA experienced two consecutive quarters of declines in GDP in the second and third quarters of 2020. It even recorded its steepest quarterly drop in the second quarter of 2020.^[^ ^12^ ^]^ What is even worse is that the second wave of COVID‐19 has sent many European countries back into lockdown, with a continuous increased death toll.

The nationwide or city‐wide coronavirus testing has been approved to be an efficient way to screen out the infectious entities from public, thus cutting off the source of transmission. Successful countries in doing so include Australia, Singapore, and China.^[^ ^13^, ^14^, ^15^ ^]^ The most widely used detection technique is the polymerase chain reaction (PCR),^[^ ^16^, ^17^, ^18^ ^]^ which shows a high specificity. However, the high testing rate requires lots of specially trained personnel and detection kits, which are costly. In addition, it takes hours to obtain the results, which is also time‐consuming. Most significantly, PCR test suffers from false‐positive cases due to sample cross‐contamination and the fact that the detection of viral nucleic acid does not definitely associate with infectivity. Therefore, protein tests such as those used to detect antibodies in patient samples are used as complementary tests to confirm the testing results.^[^ ^19^, ^20^, ^21^ ^]^

Optical methods for virus detection have been widely explored recently due to the advantages of fast, label‐free, reusable, affordable, and portable for point of care (POC) deployment.^[^ ^22^, ^23^, ^24^, ^25^ ^]^ The noninvasive optical methods also preserve the viability of viruses, potentially applicable for the study of the transmissibility, virulence, evolvability, and immunology of virus. For instance, epitope variation could result in escape mutants that are selected under immune pressure to render a potential vaccine inefficacious. Also, optofluidic methods fuse the state‐of‐the‐art techniques in nanophotonics and microfluidics to enable a faster and real‐time detection with a low sample consumption.^[^ ^26^, ^27^, ^28^, ^29^ ^]^

In this review, we provide an overview of current on‐chip optical methods for the detection of various viruses, such as SARS, H1N1, MERS, Zika, and COVID‐19. This article focuses on three distinct aspects, i.e., flow‐free optical detection, optofluidic approaches, as well as surface‐modification‐assisted optical methods. We review the optical mechanisms of those methods and summarize critical characteristics such as limit of detection (LOD) and detection time. Future perspective of current cutting‐edge techniques in nanophotonics that might be implemented for virus detection is also discussed.

## 2 On‐Chip Optical Methods

**Figure 1:**
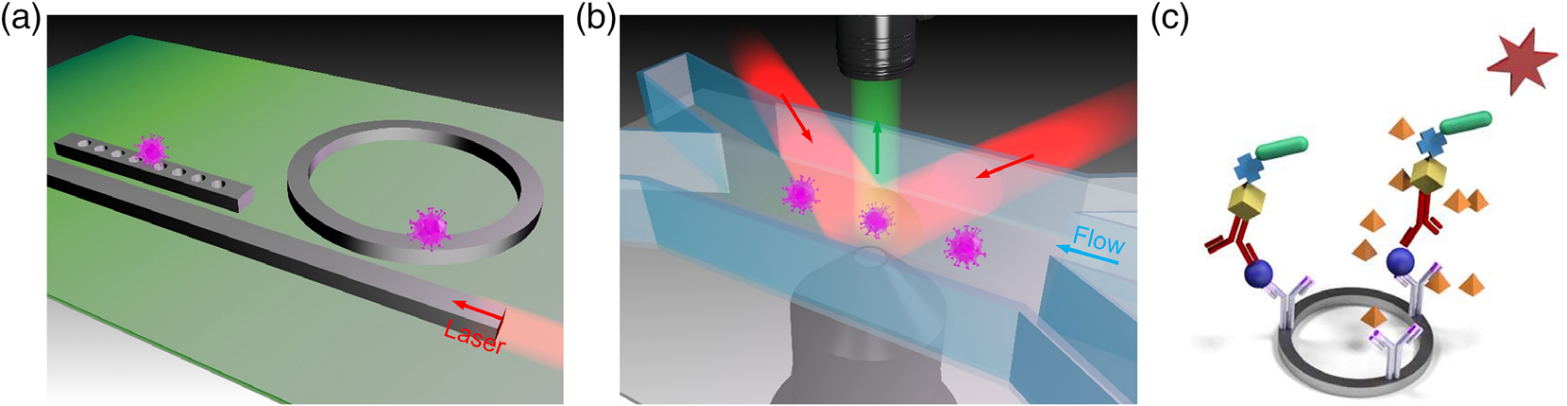
On‐chip optical detection of viruses. a) Flow‐free optical detection. b) Optofluidic detection. c) Other detection methods including the sandwiched molecule structures.

Many conventional flow‐free optical detecting methods originate from the fast development of structural design in silicon photonics, for instance, nanocavities and resonators as shown in **Figure** **1**a. The 1D photonic crystal couples light from the bus waveguide to the nanocavity. The strong resonance of nanocavity generates a hugely enhanced optical field, which exerts a strong optical force^[^ ^30^, ^31^, ^32^ ^]^ to trap virus into the nanocavity.^[^ ^33^ ^]^ When a virus is trapped, the resonance of the nanocavity will be slightly shifted, which can be detected by a spectrometer. Meanwhile, the trapped virus also allows multi‐parameter analysis of single virions, such as heterogeneity in transmission and infection.^[^ ^34^ ^]^ Viruses can also be quantified by observing the accumulated signals from multiple viruses trapped. The ring resonator is another alternative configuration that stores light in the whispering gallery mode (WGM) with a high quality factor (up to 10^6^). However, the exerted optical force is much weaker than those in nanocavities, and incapable to directly trap single viruses. In such a case, one can either rely on the free diffusion of virus to the surface of the ring resonator or modify the surface with specific receptors to assist the immobilization of virus on the surface. The output signal will change when the effective refractive index of the medium is disturbed by the existence of viruses. The viruses can also be quantified by the shifting of accumulated signals. Apart from nanostructures, many other flow‐free optical methods are also well developed, for instance, surface plasmon resonance (SPR), surface enhanced Raman scattering (SERS), multimode interference (MMI),^[^ ^35^, ^36^ ^]^ fluorescence staining, etc. These methods will be reviewed extensively in the following section.

The synergy of nanophotonics and microfluidics to fuse advantages from both fields empowers many possibilities in optical detection and manipulation. The robust conveyer of viruses in the delicate microchannels boosts the detection speed and enables real‐time diagnosis. For instance, single viruses can be confined in the hydrodynamic focusing of the flow cytometry as shown in Figure 1b. The input laser interferometry creates a detection matrix in the microchannel, whereby viruses passing by can be detected, for instance, by the differential heterodyne interferometer. Using the immunofluorescence labeling of magnetic beads, single viruses can be detected in high throughput. Many other optofluidic methods have been proposed such as optical fiber, plasmonic sensor, etc.

Other optics‐assisted methods include surface modification (SM). For instance, the sandwiched molecule structures are used to enhance the detecting signal and increase LOD as shown in Figure 1c. The photonic nucleic acid amplification has recently been used to tackle the COVID‐19 pandemic. On‐chip reverse‐transcription quantitative PCR (RT‐qPCR), a version of nucleic acids amplification reaction (NAAR), becomes a preferred method of coronavirus test due to the amplified signal in isolated unit cells on portable photonic chips. Meanwhile, microfluidic approaches have also shown great potential in virus isolation and detection by the assistance of optics and electronics. The characteristics of different optical methods for virus detection is shown in **Table** **1**.

**Table 1:** Characteristics of different on‐chip optical methods for virus detection

Technique		Virus	LOD/Sensitivity	Speed	Reference
Flow‐free optical detection	SPR	Hepatitis B	1 pg mL^−1^ of anti‐HBsAg antibody	≈10 min	[44]
	LSPR	Norovirus	95.0 copies mL^−1^	1 min	[70]
	WGM	Influenza A	5.2 × 10^−16^ g	Seconds	[50]
	Interferometry	HSV‐1	850 particles mL^−1^	1 h	[55]
	SERS	Adenovirus, rhinovirus, HIV	100 PFU mL^−1^	30–50 s	[92]
	MMI	H1N1, H2N2, H3N2	Single	Real time (excl. sample preparation)	[36]
	Metamaterials	MS2, PRD1	1 μm^−2^	Real time	[96]
	Fluorescence	Human cytomegaloviruses	Single	Real time (excl. sample preparation)	[81]
	Optical trapping	H5N1	16−19 pM for gene sequences	Real time (excl. sample preparation)	[84]
	Nanowires	HBV	2 copies per reaction	2–3 h	[83]
Optofluidics	Flow cytometry	Dengue	10^3^ PFU mL^−1^	40 min	[115]
	Optical trapping	HIV‐1	Single	4 min	[34]
	Interferometry	Sinbis, HIV, influenza	Single	Flow velocity: 250 μm s^−1^	[103]
	Nanoplasmonics	Vesicular stomatitis virus	<10^5^ PFU mL^−1^	Real time (excl. sample preparation)	[102]
	Optical fiber	Cowpea chlorotic mottle virus	Single	Tens of seconds	[106]
	Carbon nanotubes	Rhinovirus, influenza virus, parainfluenzavirus	10^2^ EID_50_ mL^−1^	Several minutes	[89]
Other optics assisted methods	On‐chip PCR	SARS‐CoV‐2	1.8 copies per reaction	2.5–3.5 h	[146]
	SM: microring	Bean pod mottle virus	10 ng mL^−1^	45 min	[120]
	SM: plasmonics	EBOV	220 fg mL^−1^	2 h	[128]

The flow‐free optical‐detection methods focus on the utilization of advances in photonics to probe the signal variations caused by the different optical properties of viruses. The volume of detection is normally quite small, e.g., in nanoliter or picoliter. The detection LOD depends on the optical approach itself when no signal amplification strategy from the virus modification is implemented. The optofluidic methods fuse the microfluidic and optical systems to transport viruses to the detection region. These methods are requested to have capabilities of fast and sensitive signal outputs, correlating with a relatively high flow rate up to μL min^−1^. Strategies such as fluorescence labeling can be adopted to enhance the signal from the high‐speed flow stream. Apart from those methods, some biomedicine‐dominated techniques also use optics as auxiliary tools to help to detect viruses, such as on‐chip PCR and SMs. Those methods seek for the interactions of biomolecules and use optical techniques to boost the detection speed and push down the LOD of conventional methods, such as PCR and enzyme‐linked immunosorbent assay (ELISA).

## 3 Flow‐Free Optical Detection

**Figure 2:**
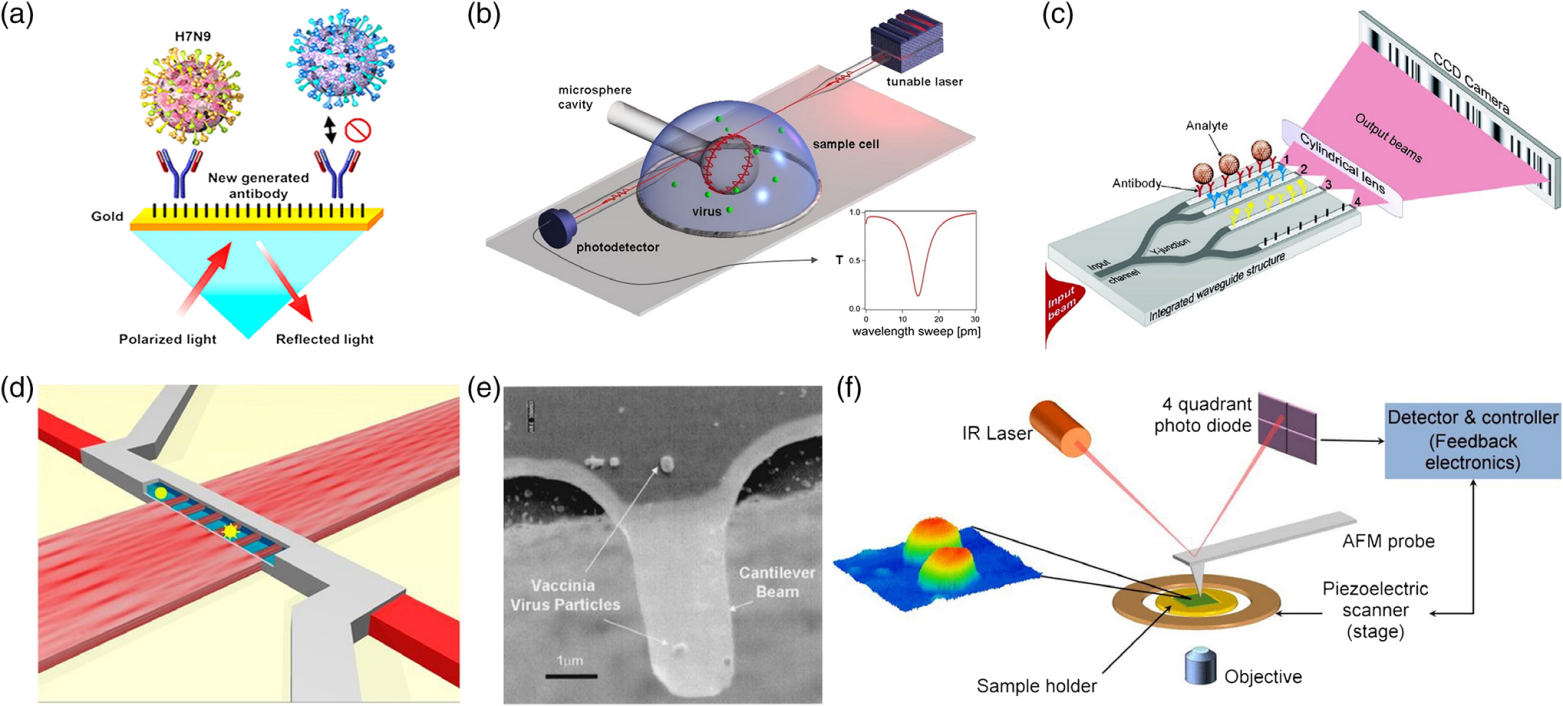
Optical detection of viruses using a) SPR methods; b) microsphere cavity; c) Young interferometer; d) MMI sensor. e) Mass detection of single viruses using micro‐cantilever beams. f) Physical characterization of height, area, and volume using the AFM. (a) Reproduced with permission.^[^ ^39^ ^]^ Copyright 2018, American Chemical Society. (b) Reproduced with permission.^[^ ^50^ ^]^ Copyright 2008, National Academy of Sciences. (c) Reproduced with permission.^[^ ^55^ ^]^ Copyright 2007, American Chemical Society. (d) Reproduced with permission.^[^ ^36^ ^]^ Copyright 2015, National Academy of Sciences. (e) Reproduced with permission.^[^ ^59^ ^]^ Copyright 2004, AIP. (f) Reproduced with permission.^[^ ^60^ ^]^ Copyright 2014, Elsevier.

Following the pioneer works on gas detection and biosensing in 1980s,^[^ ^37^, ^38^ ^]^ SPR sensors have manifested themselves as versatile and almost the most widely used biochemical sensors due to the great characteristics in sensitivity, biocompatibility, easy implementation, etc. The principle of SPR sensor relies on the propagation of excited surface plasmons along the interface of a thin metal (e.g., gold) and a dielectric (aqueous medium for virus detection) layers by the total internal reflection of the incident wave as shown in **Figure** **2**a.^[^ ^39^ ^]^ The surface of the metal layer can also be modified by coating with different antibodies targeting on specific viruses to immobilize them onto the surface.^[^ ^6^, ^7^, ^40^ ^]^ Each bound compound such as ligand and virus on the surface will shift the output signal, enabling the measurements of the binding affinity and virus quantity. To date, many SPR‐based toolkits or commercial systems have been developed to detect different viruses, such as Ebola,^[^ ^41^ ^]^ H5N1,^[^ ^42^ ^]^ infectious bursal disease virus (IBDV),^[^ ^43^ ^]^ hepatitis B virus (HBV),^[^ ^44^ ^]^ COVID‐19,^[^ ^45^ ^]^ etc. Detailed information of SPR sensors can be found in many other comprehensive reviews focused on this specific topic.^[^ ^40^, ^46^, ^47^, ^48^, ^49^ ^]^ Another classical sensor is based on the WGM of the ring resonator or microspheres as shown in Figure 2b, in which discrete changes in the resonance frequency of WGM in the microsphere occur when the surface of the ring is bound with the virus.^[^ ^50^ ^]^ Vollmer et al. reported the detection of the size and mass (5.2 × 10^−16^ g) of an Influenza A virus from the shift of resonance. He et al. developed a microlaser using WGM to detect individual polystyrene nanoparticles with 15 nm in radius, gold nanoparticles (10 nm), and influenza A virions in air, as well as polystyrene nanoparticles (30 nm) in water.^[^ ^51^ ^]^ With the naturalization of nanofabrication techniques, the WGM‐based sensors are now becoming more compact and portable for rapid POC diagnosis.^[^ ^52^, ^53^, ^54^ ^]^ This technique for virus detection relies on the virus diffusion onto the surface, which hurdles the LOD in aqueous medium because most viruses might not be bound. This limitation can be overcome by the assistance of SM with antibodies, which, however, requires specially trained personnel and compromises the detection time. An integrated optical Young interferometer sensor was proposed by Ymeti et al. for the direct, ultrasensitive, and real‐time detection of herpes simplex virus type 1 (HSV‐1) as shown in Figure 2c.^[^ ^55^ ^]^ The detection limit is quite promising at a concentration of 850 particles per mL in serum. Different from the microring resonators or microsphere cavities, the Young interferometer sensor requires a charge‐coupled device (CCD) camera instead of a spectrometer, and is capable of an extremely high sensitivity, short response time, and multiplexing simultaneously. It is anticipated to be a strong candidate for a POC viral diagnostic kit. A MMI waveguide can generate wavelength‐dependent spot patterns within the entire visible spectrum to facilitate the multiplexed detection of single biomolecules as shown in Figure 2d. Distinct targets are identified by time‐dependent fluorescence signals without the requirement of spectral demultiplexing. This method has been widely used to detect different influenza A viruses, i.e., H1N1, H2N2, and H3N2 by labeling them with different colors.^[^ ^35^, ^36^, ^56^, ^57^, ^58^ ^]^ The mass of individual viruses can be detected using silicon cantilever beams with a nanoscale thickness as shown in Figure 2e. The cantilever beam was fabricated with a dimension of 4–5 mm in length, 1–2 mm in width, and 20–30 nm in thickness.^[^ ^59^ ^]^ Based on the change in resonant frequency with the relation of virus particle mass, single vaccinia virus particles were measured with an average mass of 9.5 fg. However, this method measures only the dry mass of viruses, whereas virus study is commonly performed in aqueous medium. The sizes of viruses could be extracted and compared using the atomic force microscopy (AFM) to distinguish different virion species as shown in Figure 2f.^[^ ^60^ ^]^ Other mechanisms such as capsid–genome interactions, morphological changes that drive viral uncoating and maturation can also be unveiled by the AFM.^[^ ^61^, ^62^, ^63^ ^]^

**Figure 3:**
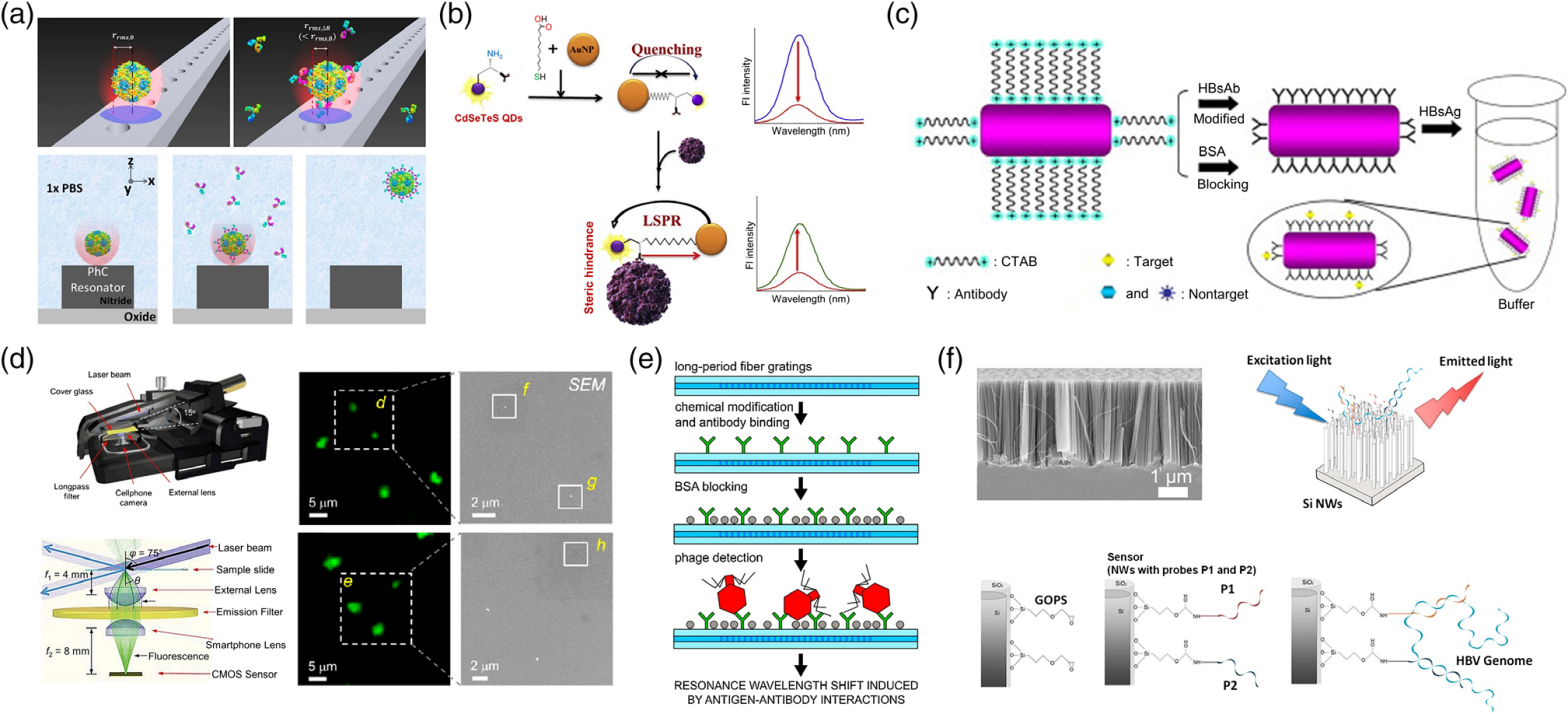
a) Measurement of stoichiometry of antibody‐binding interactions using the optical trapping in photonic crystal cavity. Optical detection of viruses using b) gold nanospheres and c) gold nanorods based on LSPR. d) Fluorescence imaging of single viruses using a cell‐phone‐based optical device. e) Long‐period fiber grating sensor for detection of viruses. f) Ultrasensitive genome detection based on silicon nanowires optical biosensors.^[^ ^83^ ^]^ (a) Reproduced with permission.^[^ ^67^ ^]^ Copyright 2015, Springer Nature. (b) Reproduced with permission.^[^ ^70^ ^]^ Copyright 2008, Elsevier. (c) Reproduced with permission.^[^ ^71^ ^]^ Copyright 2010, Elsevier. (d) Reproduced with permission.^[^ ^81^ ^]^ Copyright 2013, American Chemical Society. (e) Reproduced with permission.^[^ ^82^ ^]^ Copyright 2017, Elsevier. (f) Reproduced with permission.^[^ ^83^ ^]^ Copyright 2018, American Chemical Society.

Optical forces^[^ ^64^, ^65^, ^66^ ^]^ from the strong resonance of cavities in photonic crystals trap single viruses easily from the aqueous medium. Optical trapping has the advantage of noninvasive and is capable to study the HIN1 influenza virus and measure the stoichiometry of antibody‐binding interactions using near‐field light‐scattering technique. It was reported that an H1N1 influenza virus was approximately bound with 26 ± 4 anti‐influenza antibodies as shown in **Figure** **3**a.^[^ ^67^ ^]^ More detailed discussion about the advances in optical manipulation of biomolecules can be found in recently published review articles.^[^ ^68^, ^69^ ^]^ Metallic nanoparticles such as gold nanosphere^[^ ^70^ ^]^ (Figure 3b) and nanorod^[^ ^71^ ^]^ (Figure 3c) utilize localized surface plasmon resonance (LSPR) to detect viruses. LSPR arises from the collective oscillations of free electrons in nanoparticles with size comparable to the exciting wavelength.^[^ ^72^, ^73^, ^74^, ^75^ ^]^ The accompanied sharp peaks in scattering and absorption spectra as well as strong near‐field electromagnetic enhancements make it an excellent candidate for biosensors.^[^ ^76^, ^77^, ^78^, ^79^, ^80^ ^]^ Wei et al. developed cell‐phone‐based portable devices to image fluorescence signals from single viruses (Figure 3d). This is an encouraging step to miniaturize devices for POC deployment.^[^ ^81^ ^]^ This portable fluorescence microscopy attachment weighed only ≈186 g and could be used for designated and sensitive imaging of single viruses, providing a valuable platform for practical field activities. Other nanophotonics‐assisted potentially portable platforms include optical fiber gratings, which detects T7 bacteriophage with a concentration lower than 5 × 10^3^ PFU mL^−1^ (Figure 3e).^[^ ^82^ ^]^ Recently, Leonardi et al. developed an ultrasensitive label‐free genome‐detection system without the need of PCR, utilizing the cooperative hybridization of silicon nanowires,^[^ ^83^ ^]^ which selectively captured the optical emission of quantum confined carriers and DNA. Tested with HBV, the system achieved the LOD of 2 and 20 copies for the synthetic genome and the genome extracted from human blood, respectively, showing a better performance than the method of gold‐standard real‐time PCR in the genome analysis.

**Figure 4:**
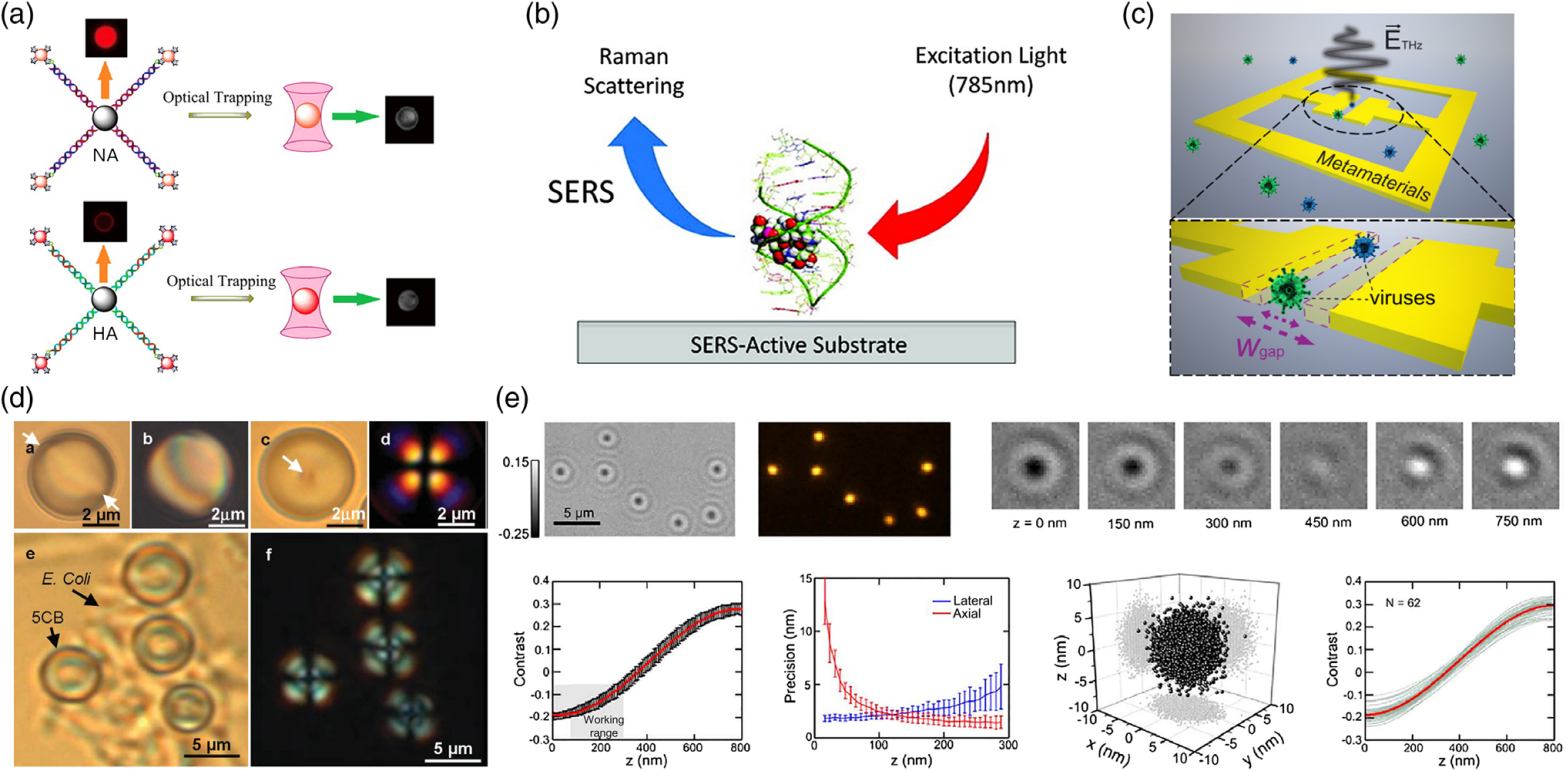
a) Optical tweezing fluorescence detection of H5N1 virus gene sequences using two‐photon excitation. b) Detection of viral nucleoprotein binding using SERS. c) Metamaterial detection of multiple viruses based on spectra shifting. d) Virus detection based on the emulsion of monodispersed LC. e) Fast and high‐resolution clarification of virus–receptor interactions using a coherent bright‐field microscope. (a) Reproduced with permission.^[^ ^84^ ^]^ Copyright 2016, American Chemical Society. (b) Reproduced with permission.^[^ ^87^ ^]^ Copyright 2012, American Chemical Society. (c) Reproduced with permission.^[^ ^96^ ^]^ Copyright 2017, OSA. (d) Reproduced with permission.^[^ ^98^ ^]^ Copyright 2009, Wiley‐VCH. (e) Reproduced with permission.^[^ ^99^ ^]^ Copyright 2017, American Chemical Society.

Apart from photonic crystals, optical tweezers with highly focused beam in free space was also implemented for the fluorescence detection of gene sequences in H5N1 virus using two‐photon excitation (**Figure** **4**a). Using the heterogeneous enrichment strategy that involves polystyrene microsphere and quantum dots, the LOD for both hemagglutinin and neuraminidase gene sequences are 16–19 pM, which is approximately one order of magnitude lower than other fluorescence‐based analysis methods.^[^ ^84^ ^]^ Raman spectroscopy has been widely used for the detection of bioparticles, such as cells and bacteria with relatively large sizes.^[^ ^85^, ^86^ ^]^ Recently, SERS has been advanced to detect single viruses.^[^ ^87^, ^88^, ^89^, ^90^, ^91^, ^92^, ^93^ ^]^ The binding of viral nucleoprotein to a polyvalent anti‐influenza aptamer can be detected by monitoring the SERS signals from the aptamer–nucleoprotein complex (Figure 4b).^[^ ^87^ ^]^

Metamaterials are delicately engineered structures with exotic electromagnetic properties, which enable to freely tailor the frequency, phase, amplitude as well as polarization.^[^ ^94^, ^95^ ^]^ Biosensors using metamaterials are typically established based on two sensing principles: 1) The shift of the resonance frequency in the transmission or reflection spectrum; 2) The characteristic absorption in the spectrum. The most classical strategy is taking the advantage of the dielectric constant or refractive index of viruses that can shift the transmission or reflection peak or dip in the spectrum. Park et al. reported a highly sensitive virus‐detection method using traditional split‐ring resonators with various capacitive gaps in the terahertz regime (Figure 4c).^[^ ^96^ ^]^ They realized the sensing of single PRD1 and MS2 viruses based on the shift in the spectra. A metamaterial biosensor based on the H‐shaped graphene resonator was manufactured and characterized by Keshavarz et al., which behaved highly sensitive to three subtypes of avian influenza viruses.^[^ ^97^ ^]^

Based on the emulsion of monodispersed liquid crystal (LC), Sivakumar et al. developed a versatile sensing method to detect both enveloped and nonenveloped viruses (Figure 4d).^[^ ^98^ ^]^ LCs of 4‐cyano‐4′‐pentylbiphenyl transit from a bipolar to radial configuration when in contact with Gram: lipid‐enveloped viruses (A/NWS/Tokyo/67). The LC emulsion methods were shown to detect the virus with a low concentration (down to 10^4^ pfu mL^−1^). To detect the early stage of virus affection in live cells with a high resolution and high speed, Huang et al. reported a coherent bright‐field microscope that provides the spatiotemporal resolution (Figure 4e).^[^ ^99^ ^]^ This microscope detected intrinsic scattered light via imaging‐based interferometry, allowing the motion tracking of a single vaccinia virus with a nanometer spatial precision (<3 nm) in three dimensions and microsecond temporal resolution (up to 100 000 frames per second). This unprecedented performance offers many possibilities to elucidate the virus–host interaction, which is essential for the diagnostics, vaccine, and antiviral drug developments.

## 4 Optofluidic Detection of Virus

**Figure 5:**
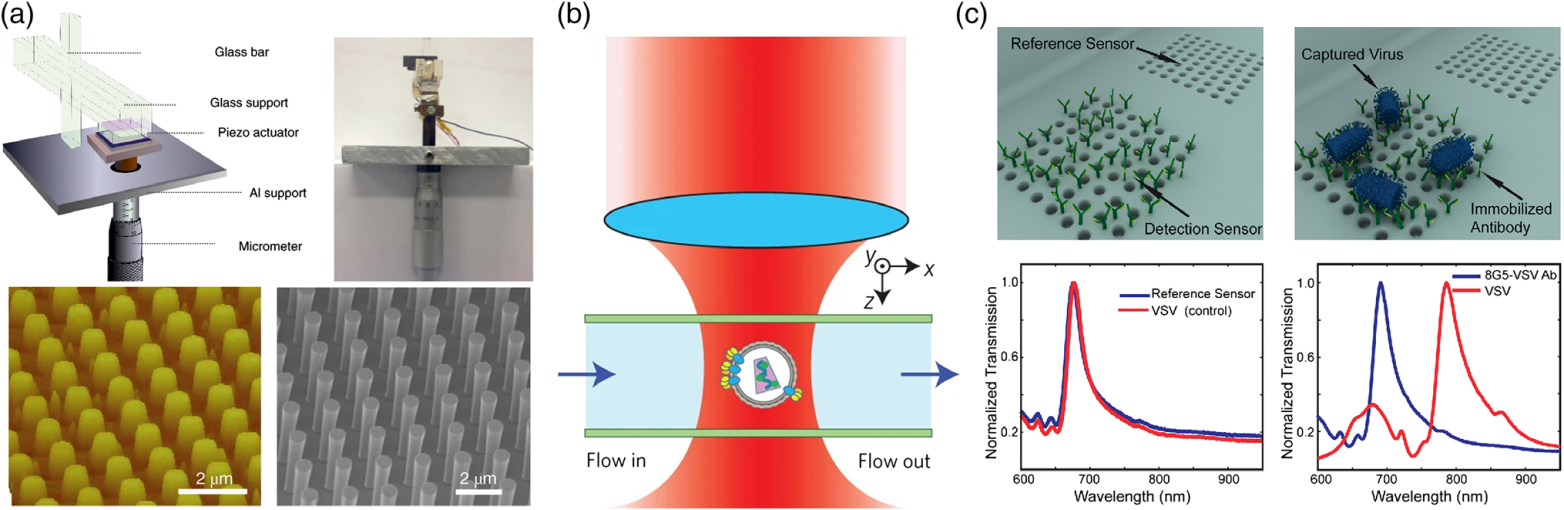
a) Biomarker detection using SERS in an optofluidic chip. b) Optical trapping of single mammalian viruses in an optofluidic chip to study the heterogeneities of virions in transmission and infection at single‐virus level. c) Optofluidic detection of viruses in a plasmonic optofluidic chip. (a) Reproduced with permission.^[^ ^100^ ^]^ Copyright 2020, Springer Nature. (b) Reproduced with permission.^[^ ^34^ ^]^ Copyright 2014, Springer Nature. (c) Reproduced with permission.^[^ ^102^ ^]^ Copyright 2010, American Chemical Society.

Optofluidics fuses two state‐of‐the‐art research fields, nanophotonics and microfluidics, thereby exhibits unparallel advantages from each technique to realize novel and powerful functionalities. It is a paradigm to transport single bioparticles such as viruses to the detection area in the microchannel and use various optical approaches to detect them. The assistance of fluid boosts the detection speed and enables real‐time diagnosis of viruses. Developing high‐sensitive and fast optofluidic detection device is in urgent demand nowadays due to frequent outbreaks of pandemics worldwide in the past two decades. A good example is the development of an optofluidic device for rapid and label‐free detection of biomarkers using gold‐coating nanopillar enabled SERS (**Figure** **5**a).^[^ ^100^ ^]^ This chip can be performed in a flow rate of 1.2 μL h^−1^. By implementing optical tweezers in a microchannel, single viruses can be trapped and analyzed. Pang et al. reported that the trapping of individual HIV‐1 viruses allowed the multi‐parameter analysis of single virions in culture fluid environmentally mimicking native conditions (Figure 5b).^[^ ^34^, ^101^ ^]^ It was shown that individual HIV‐1 differed in the number of envelope glycoproteins by over one order of magnitude, indicating substantial heterogeneities of viral particles in transmission and infection at the single‐virus level. Using the plasmonic nanohole array in an optofluidic chip, Yanik et al. reported a fast and label‐free virus‐detecting chip utilizing group‐specific antibodies targeting highly divergent strains of rapidly evolving viruses (Figure 5c).^[^ ^102^ ^]^ The large‐enveloped‐DNA viruses (vaccinia virus) and small‐enveloped‐RNA viruses (pseudotyped Ebola and vesicular stomatitis virus) were detected with ultra low noise signal ratio and a high dynamic range in three orders.

**Figure 6:**
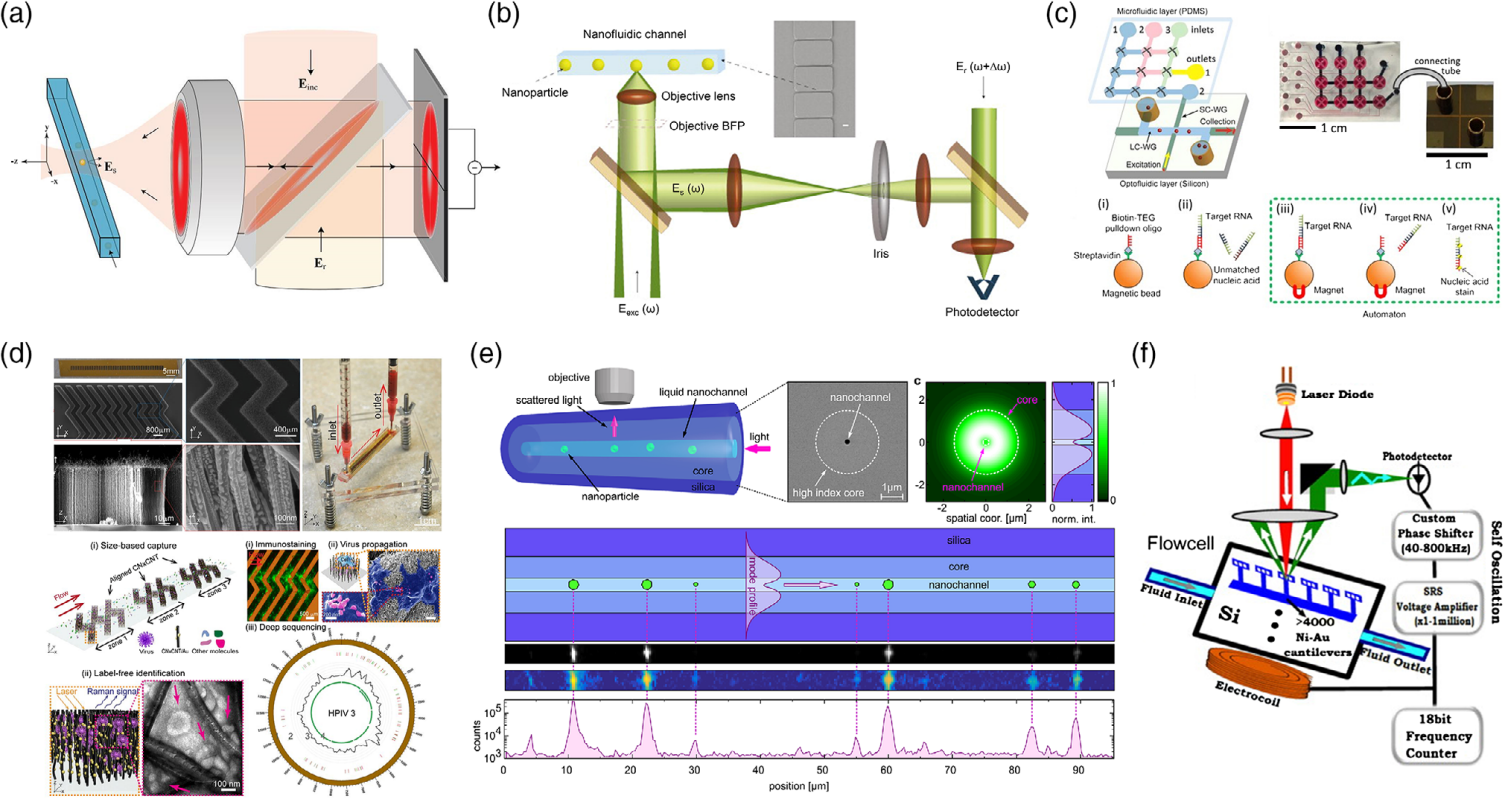
a) Detection, sizing, and sorting of viruses using optofluidic heterodyne interferometric detection of the light scattering. b) HIV virus and bacteriophage detection using the heterodyne interferometry and dark‐field microscopy. c) Single nucleic acid fluorescence detection of Ebola viruses in liquid‐core optical waveguides on a silicon chip. d) Rapid enrichment and optical identification of viruses using carbon nanotube arrays with differential filtration porosity in a portable microfluidic system. e) Fast tracking of viruses in a hollow optical fiber with nanofluidics. f) Detection of Hepatitis A and C viruses using MEMS cantilever actuated system. (a) Reproduced with permission.^[^ ^103^ ^]^ Copyright 2010, American Chemical Society. (b) Reproduced with permission.^[^ ^104^ ^]^ Copyright 2012, Elsevier. (c) Reproduced with permission.^[^ ^105^ ^]^ Copyright 2015, Springer Nature. (d) Reproduced with permission.^[^ ^89^ ^]^ Copyright 2020, National Academy of Sciences. (e) Reproduced with permission.^[^ ^106^ ^]^ Copyright 2015, American Chemical Society. (f) Reproduced with permission.^[^ ^109^ ^]^ Copyright 2011, Elsevier.

Optofluidic systems integrated with the heterodyne detection were used to detect, sort, and measure the sizes of single viruses as shown in **Figure** **6**a.^[^ ^103^ ^]^ The heterodyne detection eliminated phase variations from trajectories of different particles to improve the recognition accuracy, showing great potential in the screening and detection of different viruses (e.g., Sindbis and HIV). Later, Mitra et al. combined the heterodyne interferometry with dark‐field microscopy for HIV virus and bacteriophage detection (Figure 6b),^[^ ^104^ ^]^ down to the size of 20 nm in high sensitivity and precision. It also resolved distribution of nanoimpurities (≈20–30 nm) in clinically relevant media. To reduce the sample preparation time and reduce the potential contamination, Cai et al. proposed an optofluidic chip that could implement sample preparation and target preconcentration on a polydimethylsiloxane‐based optofluidic chip (Figure 6c).^[^ ^105^ ^]^ The single nucleic acid fluorescence detection of viruses was conducted within liquid‐core optical waveguides on a silicon chip in 10 min with a good specificity, an LOD of 0.2 pfu mL^−1^, and a dynamic range of thirteen orders of magnitude. This hybrid system provides a step toward the portable instruments by including the procedure of sample preparation, which drives it with a high potential in the POC diagnosis. Recently, a fast virus capture, concentration, and detection platform was proposed with carbon nanotube arrays (Figure 6d),^[^ ^89^ ^]^ which achieved a fast detection of influenza A viruses within a few minutes using as little as 10^2^ EID_50_ mL^−1^ (50% egg infective dose per microliter), providing a virus specificity of 90%. It was also capable of sequencing and could be used for timely monitoring of viral outbreaks. To improve the limit of measurement speed by the fluorescence emission lifetime and duration by the photobleaching and thermal diffusion, Faez et al. used a hollow optical fiber containing the nanofluids to track 20 nm nonstained dielectric particles as well as individual cowpea chlorotic mottle virus virions with size of 26 nm at a rate of over 3 kHz for a duration of tens of seconds (Figure 6e).^[^ ^106^ ^]^ Hollow optical fibers were also used in many virus filtration systems.^[^ ^107^, ^108^ ^]^ Similar to the metamaterials, the microelectromechanical system (MEMS) cantilever‐actuated system could be used for detecting Hepatitis A and C viruses with dynamic range over 1000 and a limit of detectable concentration at 0.1 ng mL^−1^ or 1.66 pM (Figure 6f).^[^ ^109^ ^]^

**Figure 7:**
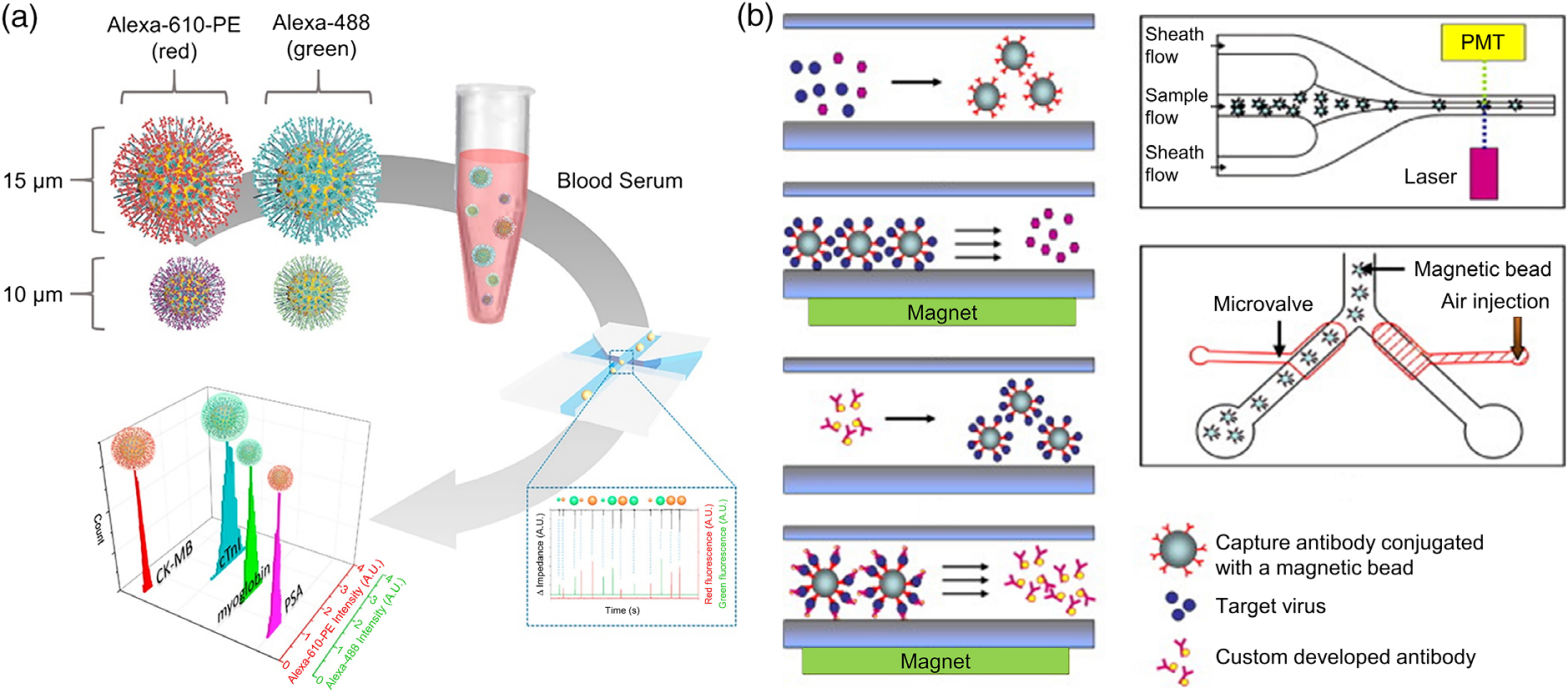
a) Schematics of flow cytometry system for biomarker detection. b) Magnetic‐bead‐assisted flow cytometry for the fast and high‐sensitive virus detection. (a) Reproduced with permission.^[^ ^111^ ^]^ Copyright 2018, Elsevier. (b) Reproduced with permission.^[^ ^115^ ^]^ Copyright 2008, Elsevier.

Flow cytometry is a powerful tool used to detect and characterize physical and chemical properties of bioparticles. The fluorescence‐activated cell sorting is widely used to sort cells and bacteria (**Figure** **7**a). A conventional flow cytometry is generally infeasible to directly measure viruses due to the weak light‐scattering signal caused by the small size of virion.^[^ ^110^, ^111^ ^]^ To facilitate the virus detection, various approaches have been used including fluorescence staining,^[^ ^112^, ^113^, ^114^ ^]^ magnetic/gold bead^[^ ^115^ ^]^ labeling (Figure 7b), and integration of advanced optical systems including dark‐field microscopy.^[^ ^116^, ^117^, ^118^ ^]^

## 5 Other Methods with Optics

### 5.1 Surface Modification

For the detection of virus based on an optofluidic chip, an optical subsystem is usually set up for readout digital or image signal. However, the optical detection part is not specific to the target in general. Therefore, the specificity for the determination of virus target is critically important and, on the other hand, commonly depends on biorecognition elements which are usually immobilized on optical subsystem surface through SM. Sensitivity to biological agents is conferred by functionalizing the sensing surface with molecules selected against targets of interest. SM methods vary with different surface properties, which largely depend on the surface material.

A fused silica capillary optofluidic ring resonator was applied to detect and quantify filamentous bacteriophage M13 virus in aqueous.^[^ ^119^ ^]^ Anti‐M13 antibodies were first covalently attached on the ring surface coated with aminosilane to provide a bioselective layer. Then, the interior surface of ring was cleaned using a mixture of HCl–methanol and rinsed thoroughly using DI water. Next, 3‐aminopropyltrimethoxysilane in water was passed through for 20 min to silanize the surface. After rinsing with DI water, 5% homobifunctional cross‐linker glutaraldehyde in water was injected into the chip to link protein A to the aminated surface. Then, protein A dissolved in phosphate‐buffered saline buffer was introduced into the ring for 10 min. Finally, the chip was ready for the detection of M13 virus and showed an LOD as low as 1000 pfu mL^−1^.

**Figure 8:**
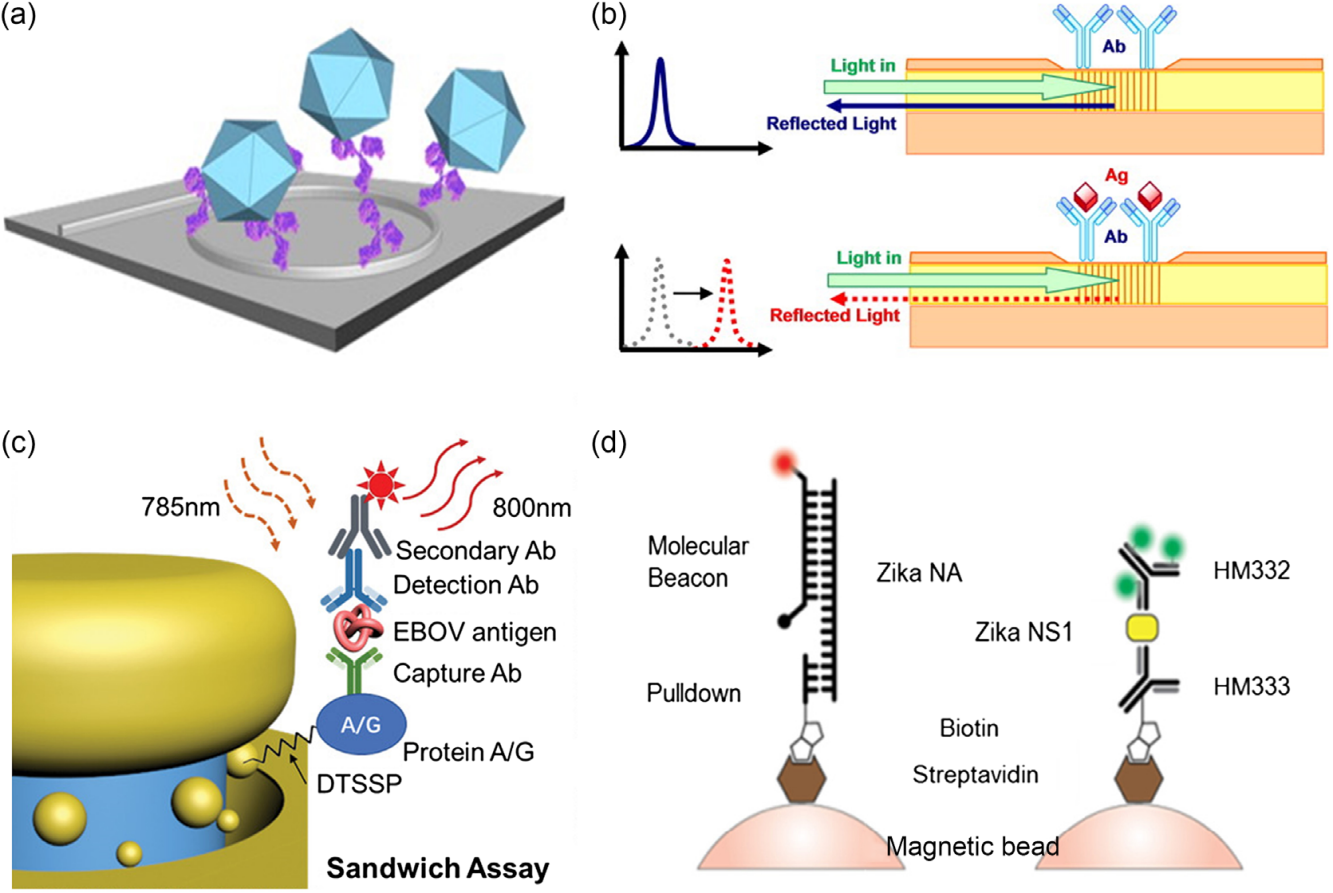
SM strategies on virus detection device. a) Virus binding to the antibodies on the microring induces changes in support optical resonance. b) Principle of the grating structure to detect MS2 virus. c) The sandwich structure of EBOV nanoantenna sensor. d) Target isolation with high specificity to detect ZIKV virus.^[^ ^57^ ^]^ (a) Reproduced with permission.^[^ ^120^ ^]^ Copyright 2012, Elsevier. (b) Reproduced with permission.^[^ ^126^ ^]^ Copyright 2011, Elsevier. (c) Reproduced with permission.^[^ ^128^ ^]^ Copyright 2019, Wiley‐VCH. (d) Reproduced with permission.^[^ ^57^ ^]^ Copyright 2018, OSA.

In addition to using silica as the chip material, the applications of silicon chips in sensing was increasing. Bean pod mottle virus (BPMV), a pathogen of great agricultural importance, was detected by silicon photonic micro‐ring resonator with an LOD of 10 ng mL^−1^, as shown in **Figure** **8**a.^[^ ^120^ ^]^ To achieve specificity of the system in detecting BPMY, the chip was immobilized with monoclonal antibodies specific for BPMV by a few steps of SM. For the SM methods of silicon material, many relevant works have been reported although the target was not virus.^[^ ^121^, ^122^, ^123^, ^124^, ^125^ ^]^ Waveguides and Bragg gratings were fabricated into the silica‐on‐silicon substrates to detect *Escherichia* virus MS2, as shown in Figure 8b.^[^ ^126^, ^127^ ^]^ To facilitate the detection, surfaces were modified with an amino‐terminated silane monolayer 3‐aminopropyltriethoxysilane for 1 h and then deployed by glutaraldehyde cross‐linking for covalent attachment of recombinant protein A/G, to immobilize the sheep anti‐MS2 antibodies. When the surface‐immobilized antibodies bound with target antigens carried by the flow, the local refractive index will change, so as the output signal. The study demonstrated the selective detection of MS2 viruses without significant nonspecific binding.

Using LSPR, gold was used to form nanodisks and then plasmonic nanocavity array was used to detect Ebola virus antigen (EBOV) with a sandwich assay (Figure 8c).^[^ ^128^ ^]^ Before initiating the assay, the sensor surfaces were functionalized with a self‐assembled monolayer of 3,3′‐dithiobis (sulfosuccinimidyl propionate) to generate the thiol–gold linker, and subsequently bound with a layer of protein A/G to serve antibody anchors. Layers of self‐assembled monolayer plus protein A/G had a thickness around 6.1 nm to prevent the loss of irradiative fluorescence signals. Figure 8c shows a schematic representation of the EBOV sandwich (protein A/G–capture antibody–antigen–detection antibody–secondary antibody) bound to a nanoantenna array. The nanoantenna‐assisted‐biosensor detected EBOV‐soluble glycoprotein in serum with a concentration down to 220 fg mL^−1^, which is 240 000‐fold higher in sensitivity compared with the existing rapid EBOV immunoassay (53 ng mL^−1^). Another work also introduced *N*‐(3‐dimethylaminopropyl)‐*N*′‐ethylcarbodiimide hydrochloride and *N*‐hydroxysuccinimide to activate the modified SPR surface for EBOV detection.^[^ ^41^ ^]^

In other works, optofluidic systems with MMI multiplexing were used to detect Zika nucleic acid and protein biomarkers.^[^ ^57^, ^105^, ^129^, ^130^ ^]^ Figure 8d shows a solid‐phase extraction method using beads. Streptavidin‐coated magnetic microspheres bound with biotin‐functionalized Zika virus monoclonal antibody or biotinylated complimentary oligonucleotide formed the pulldown complex. This optofluidic biosensor based on fluorescence signals is a paradigm of highly specific and sensitive approaches for simultaneous detection of multiple molecular targets, superior to the gold‐standard techniques such as RT‐PCR and ELISA, which are limited to a single type of target.

Although there are various surface treatment methods, the common principle is to create a biorecognition layer on the chip surface through a few chemical or biological modifications. First is the surface activation with active group. Then single or a few linkers is introduced following the active group. Finally, the capture probe which has specificity determination to the target is linked.

### 5.2 Photonic Nucleic Acids Amplification for Virus Detection

In COVID‐19 pandemic, RT‐qPCR, a version of NAAR, becomes a preferred method of coronavirus test that contributes in alleviating the spread of coronavirus.^[^ ^131^ ^]^ Despite of good performance of conventional RT‐PCR, it also has some drawbacks such as long reaction time (≈2 h) and undesirable sensitivity (typically 500 copies mL^−1^), which have been evidenced in the battle against COVID‐19.^[^ ^132^ ^]^

**Figure 9:**
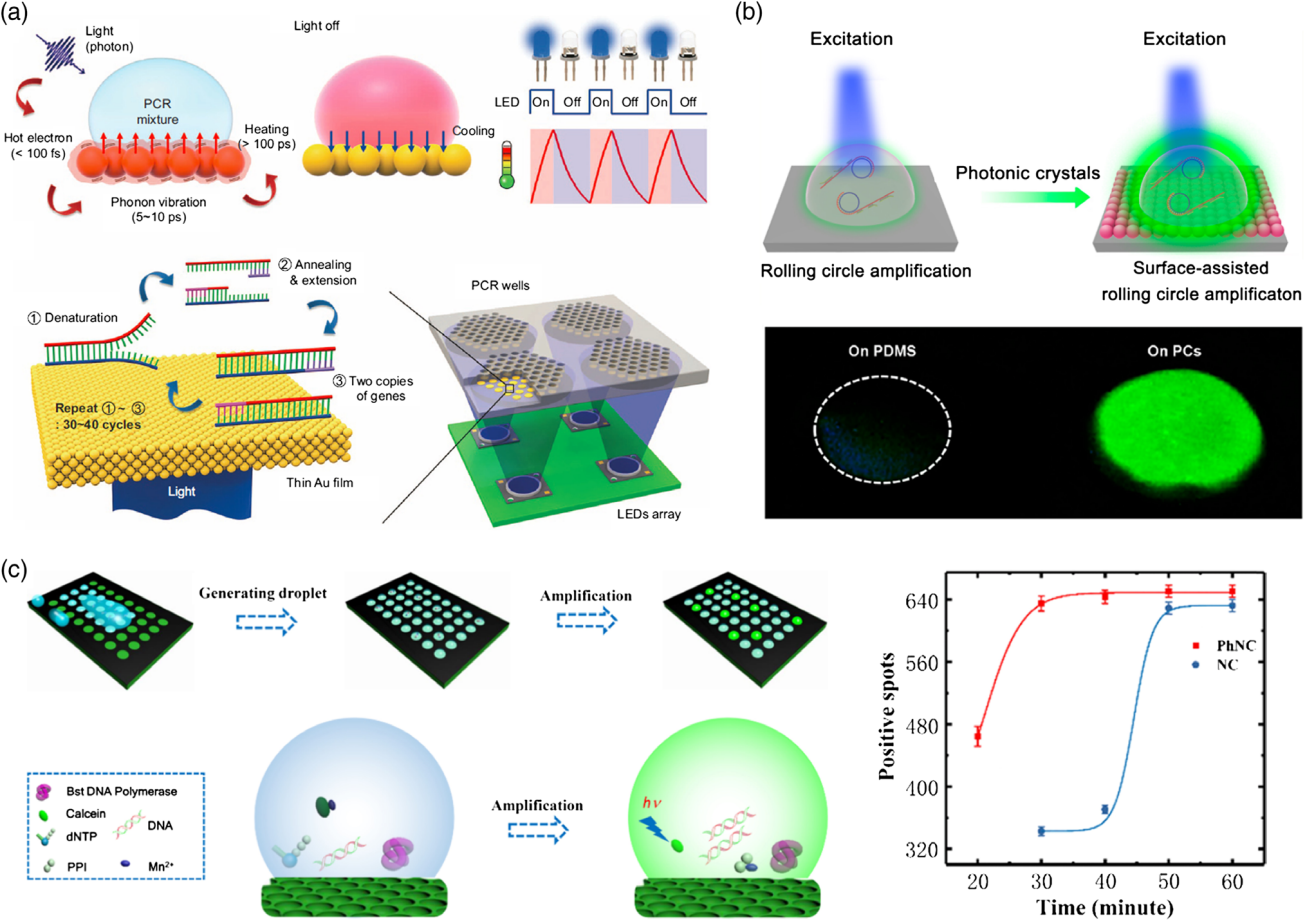
Schematic illustrations of the main photonic nucleic acids detection technologies. a) Ultrafast photonic PCR on the Au film. b) Photonic particles enhanced RNA detection. c) Photonic‐enhanced digital LAMP. (a) Reproduced with permission.^[^ ^133^ ^]^ Copyright 2015, Springer Nature. (b) Reproduced with permission.^[^ ^139^ ^]^ Copyright 2018, American Chemical Society. (c) Reproduced with permission.^[^ ^140^ ^]^ Copyright 2018, American Chemical Society.

Many photonic NAARs have been proposed before this pandemic to shorten the reaction time via accelerating thermocycling or enhancing optical signal to realize fast viral pathogen detection. These methods can be classified into photothermal PCR and photonic enhanced NAAR. In photothermal PCR, nanomaterials with photothermal effect, such as gold nanoparticles (AuNPs),^[^ ^133^, ^134^ ^]^ Au nanofilm (**Figure** **9**a),^[^ ^135^, ^136^ ^]^ and Fe_3_O_4_ NPs,^[^ ^137^ ^]^ take the place of bulk heating block in traditional PCR to heat the reaction mixture. Thanks to the larger contact area between heater and solution, lower heat capacity and high efficiency of light‐to‐heat conversion, photothermal PCR can achieve ultrafast thermocycling with the heating and cooling rates of 16.6 and 9.4 °C s^−1^, respectively.^[^ ^134^ ^]^ The 40 thermal cycles can be accomplished in 5–10 min,^[^ ^138^ ^]^ whereas it takes about 2 h for common PCR. Inspired by fluorescence signal enhancement of photonic particles (PCs) (Figure 9b), photonic‐enhanced NAAR were developed in combination of PCs with traditional NAAR, in which PCs serve as the substrate to maximize the emission signals of fluorescein in reaction mixture by slow‐photon effect.^[^ ^139^ ^]^ It can detect weak fluorescence signals of amplification products at a very low concentration, thus reducing the required reaction time.^[^ ^140^ ^]^ Take the photonic‐enhanced loop‐mediated isothermal amplification (LAMP) (Figure 9c) as an example, the detection time of infected bacteria DNA was shortened to 40 min from 60 min and the detection limits was about 10 times lower than the standard LAMP.^[^ ^141^, ^142^ ^]^ In addition, ring resonator‐based NAAR methods were also reported in multiple and label‐free nucleic acids detection.^[^ ^143^, ^144^, ^145^ ^]^

Although the photonic PCR dramatically shortens detection time, its sensitivity cannot meet the requirement of some clinical scenarios; for instance, the detection of virus in the early stage of infection^[^ ^132^ ^]^ and virus residue on objective surface,^[^ ^146^ ^]^ and thus hinders its application in clinical practice. Digital PCR (dPCR) could be helpful to overcome this problem. Through distributing targets into isolated microreactions with nanoliter or picoliter volume, dPCR can realize single nucleic acid detection after amplification but suffers from long reaction time.^[^ ^147^, ^148^ ^]^ Considering the short reaction time of photonic PCR, some groups have done preliminary work in exploring photonic digital NAAR to achieve ultrafast and ultrasensitive infective pathogens detection.^[^ ^140^, ^149^ ^]^ Photonic digital NAAR with the merits of high sensitivity and short reaction time show bright prospective in POC diagnosis and could be a powerful technology in response to the next virus outbreak.

## 6 Outlook and Discussions

Clinical utilities of on‐chip optical methods need to assess many critical factors, such as speed, volume, cost, LOD, heterogeneity, etc. Optical methods are fast with detection time from milliseconds to several hours. However, most flow‐free detection methods can only detect samples of small volumes, normally in picoliter or nanoliter. Meanwhile, many viruses have similar optical properties (e.g., mass, refractive index), imposing great difficulties in the detection of virion heterogeneity using these methods. The commercial SPR machine can be operated at a flow rate of several μL min^−1^, but the prices of machine and each chip cost approximately hundreds of thousands of US dollars and a few hundred US dollars, respectively. Moreover, the reagent solutions may also cost hundreds to thousands of US dollars. The nanoplasmonic platform may serve as a paradigm for the virus detection with the assistance of antibody either in a single layer or at a sandwiched molecule structure. The cost is also acceptable due to the standard sample fabrication and surface treatment processes. Another fast, sensitive, and promising approach is the photonic digital NAAR, which detects high‐heterogeneity virions with affordable expenses, suitable for conducting experiments in almost all virion‐accessible hospitals and labs.

Recent advances in nanophotonics have made huge progress in the sensing of biomolecules. All‐dielectric asymmetric metasurfaces with the intriguing physics of bound states in the continuum, have unprecedented feature of high‐quality resonances, which provide an ultrasensitive label‐free analysis platform for biosensing.^[^ ^150^, ^151^, ^152^, ^153^, ^154^, ^155^ ^]^ Yesilkoy et al. reported a metasurface to achieve an LOD of three molecules per μm^2^ by using a pixel‐based thresholding method.^[^ ^150^ ^]^ An imaging‐based nanophotonic device was proposed recently for the detection of molecular fingerprints using a 2D pixelated dielectric metasurface.^[^ ^152^ ^]^ Each pixel was tuned to a discrete frequency, and its amplitude would be affected by the presence of biomolecules.^[^ ^151^ ^]^ The affected spectrum was then translated into a barcode‐like absorption map. This cutting‐edge nanophotonic technique resolved absorption fingerprints without the requirement of spectrometry, being potentially applicable for detecting species of viruses. Park et al. reported the observation of exceptional point (EP) in a plasmonic system by hybridizing detuned resonances in multilayered plasmonic structures.^[^ ^156^ ^]^ The EP system pushed up the detection sensitivity of anti‐immunoglobulin G to 4821 nm per RIU and stipulated an LOD of 15 × 10^−12^ g L^−1^, which outperformed previous plasmonic array sensors. High‐order EP systems were also found to enhance the sensitivity.^[^ ^157^ ^]^ Meanwhile, Jing et al. used the enhanced mode splitting and a strongly modified transmission spectrum to enhance the sensitivity of the WGM sensor.^[^ ^158^ ^]^ Their device did not require complex materials, hybrid systems, optical gain, or low temperatures. Those progresses in extreme photonic systems will enable future advances in faster and more sensitive biosensing.

Comparing with gold‐standard nucleic acid assays using PCR testing and typical ELISA, optical methods empower a faster, label‐free and single‐virus detection, providing alternative solutions to tackle current outbreak of pandemics. More opportunities in sequencing, genomic and heterogeneity analysis, viral pathogenicity, which are not attainable by conventional PCR, can also be studied using optical chips. Meanwhile, on‐chip PCR is showing more and more evidence in the improvement of detection sensitivity. Although there is still a gap between the massive commercialization and laboratory study, future simplification and increased specificity plus the intrinsic high sensitivity of photonic devices would contribute hugely to the affordable nation‐wide detection to prevent and stop pandemics. The precise control of micro‐environment conditions for drug testing in the optofluidic chip will improve the efficiency in vaccine and antiviral drug development. All these possibilities help to accelerate the development of virus diagnostics, vaccines, and antiviral drugs to tackle unknown pandemic outbreaks such as COVID‐19.

## Conflict of Interest

The authors declare no conflict of interest.
